# Computational eco-systems biology in *Tara* Oceans: translating data into knowledge

**DOI:** 10.15252/msb.20156272

**Published:** 2015-05-28

**Authors:** Shinichi Sunagawa, Eric Karsenti, Chris Bowler, Peer Bork

**Affiliations:** 1 European Molecular Biology LaboratoryHeidelberg, Germany; 2 Ecole Normale Supérieure, Institut de Biologie de l'ENS (IBENS), and Inserm U1024, and CNRS UMR 8197Paris, France; 3 Max-Delbrück-Centre for Molecular MedicineBerlin, Germany

**Figure 1:**
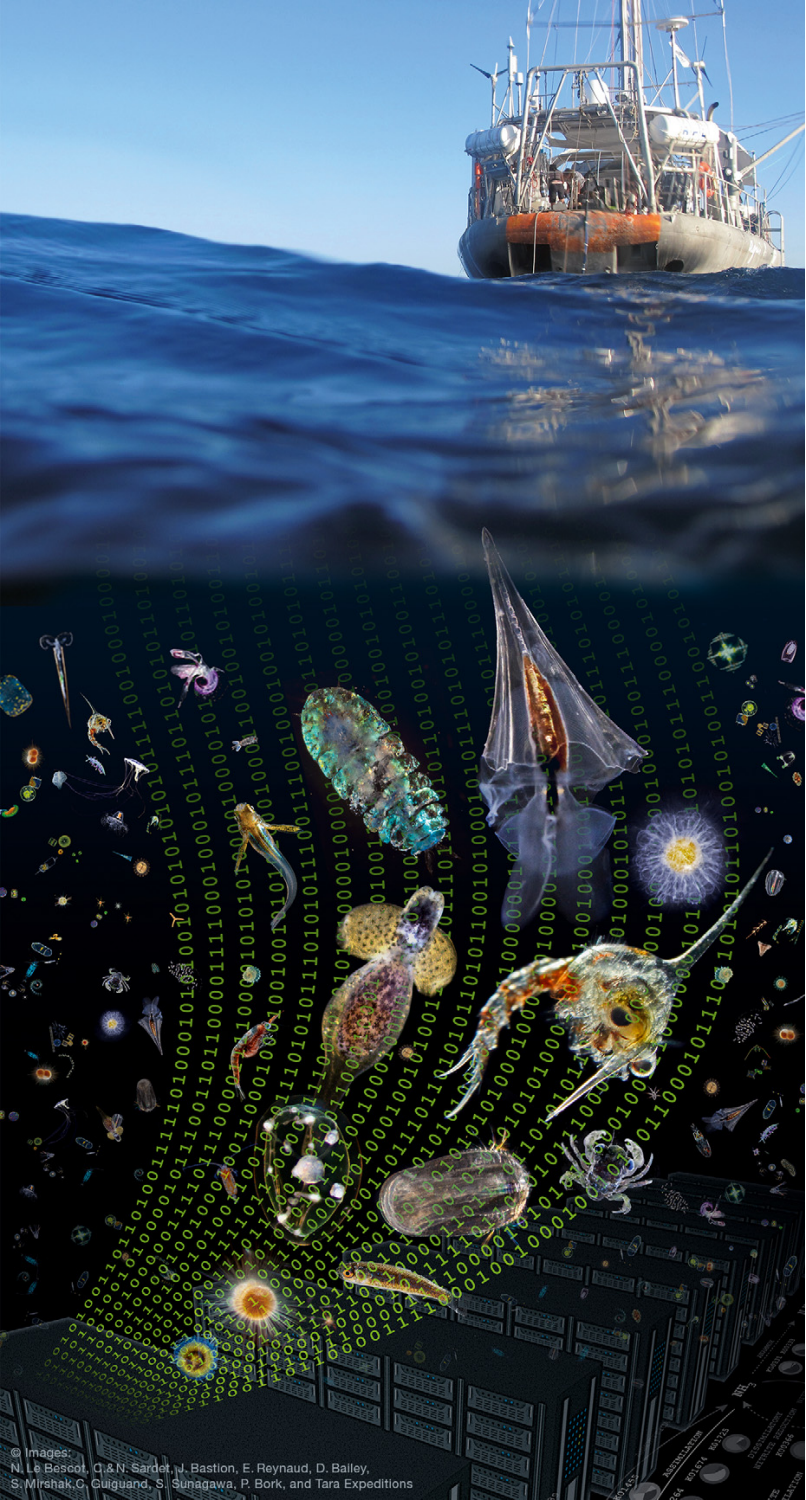
Translating *Tara* Oceans' data deluge into knowledge.

In molecular systems biology, data are flooding us at an ever-increasing pace, from genomic to transcriptomic and proteomic information, complemented by spatially and time-resolved data obtained at multiple scales. Computational biology usually integrates these layers of information at the cellular and biochemical levels. But how does the interplay between experimental and computational biology work if this information is coming not only from a cellular system, but from an entire ecosystem and if this ecosystem spans the entire Earth? Here, we illustrate some of the computational challenges and promises of large-scale eco-systems biology studies (Raes & Bork, 2008) in the context of the *Tara* Oceans project (Fig1), which has arguably been one of the wettest wet laboratory experiments ever.

From 2009 to 2013, the schooner *Tara* sampled ocean plankton spanning several orders of magnitude in size at 210 stations over a range of depths (down to 2,000 m) around the world's oceans, together with various oceanographic measures such as temperature, salinity, nutrient concentrations, as well as visual monitoring of plankton far beyond the resolution of the naked eye (Bork *et al*, 2015). This adventurous wet part of the project obviously needed a dry counterpart on land, not only mastering study design, standards (e.g. standard operational protocols), archiving and logistics (Pesant *et al*, 2015), but also the arduous yet exciting part of translating heterogeneous data into knowledge at a truly planetary scale. To tackle this grand challenge, an interdisciplinary team was formed early on to take an integrative approach and to maximize the interactions between fields and people, with the objective of promoting research that was beyond what individual laboratories could accomplish alone (Karsenti, 2015).

In total, > 35,000 samples, each one with an individual barcode and with contextual metadata, were collected for morphological, environmental and genomic analysis. Of the latter, a subset of ca. 600 samples had been prioritized early on to balance biogeographical coverage and analysis costs (Table1). At regular meetings and telephone conferences, consortium scientists were able to build a network of the different methodological approaches, biomolecular data types, diverse organism groups and oceanographic parameters, and then to superimpose a range of global and discipline-specific questions that could be addressed with these data.

**Table 1:** Taxonomic and genetic diversity analysed to date by the *Tara* Oceans project.

	Eukaryotes	Prokaryotes	Viruses
Taxonomic diversity			
Method	18S rRNA (PCR tags)	16S rRNA (_mi_tags)	Contigs (assembly)
Detected diversity	110 k OTUs	35 k OTUs	5.5 k populations
Novel taxa	23 k OTUs	ND	ND
Samples	334	139	43
Stations	47	67	26
Genetic diversity			
Method	Metatranscriptomics	Metagenomics	Metagenomics
Detected diversity	7.6 M genes^a^	40 M genes^a^	1 M proteins^b^
Novel genes	> 30%^c^	> 80%^a^	< 20%^b^
Samples	29	243	43
Stations	3	68	26

Cells highlighted in yellow: only those data are, in principle, comparable but even here station numbers and filters differ.

a Based on clustering at 95% nucleotide sequence similarity.

b Based on clustering at 60% protein sequence identity.

c Based on taxonomic assignments.

To enable synchronization of the different laboratories, the first steps of the analysis involved data standardization, normalization, quality control and public deposition of the data. For example, signal profiles that had been recorded *in situ* by numerous instruments had to be calibrated and validated, data from satellites and autonomous floats were integrated, and on land, analyses of samples added further data such as nutrients, pigments and carbonate chemistry to yield comprehensive environmental data. Digital images were analysed to extract features describing the shape and diversity of the captured organisms. Trillions of sequenced DNA base pairs were translated into organismal abundance and diversity using 16S and 18S rRNA gene data on the one hand and assembled into genes and genomes based on metagenomics and metatranscriptomics on the other hand (Jaillon *et al* in preparation; Sunagawa *et al*, 2015). For the analysis of metagenomics data alone, millions of CPU hours distributed over high-performance clusters with terabyte memory nodes were required to solve this gigantic puzzle. The public deposition of the raw and derived data was a challenge on its own, not only due to their sheer volume (to date, 11.5 terabytes), but also due to the need to contextualize and cross-link data from heterogeneous sources. But with the much-appreciated support from the European Bioinformatics Institute (EBI) and the environmental data publisher PANGAEA (www.pangaea.de), it was finally accomplished.

As a first integration milestone, a number of general resources were created: an 18S rRNA gene-based census of eukaryotic biodiversity and an ocean microbial reference gene catalogue; the latter derived from the analysis of organisms filtered by size to enrich viral and prokaryotic content. Both biomolecule-based data types give insights into the biodiversity of the world's oceans at unprecedented scale (Sunagawa *et al*, 2015; de Vargas *et al*, 2015). The resources, together with other data types (e.g. microscopy images, environmental parameters and oceanographic measurements), were then utilized to establish an overview of DNA virus distribution in the oceans (Brum *et al*, 2015), to derive global species interaction networks across all domains of life and viruses (Lima-Mendez *et al*, 2015) and to integrate oceanographic and biological data to study plankton dispersal at a major chokepoint of global ocean circulation (Villar *et al*, 2015). These studies exemplify how an ecosystems biology approach can be used to interpret molecular data in the context of planetary-scale processes such as ocean currents, temperature gradients and nutrient cycles.

Analysis of the *Tara* Oceans' data is likely to continue for years, perhaps decades. Together with other data sources and types, the *Tara* Oceans' data sets should contribute to a comprehensive parts list of organisms, genes and genomes in our oceans, although challenges in data comparability still need to be addressed (Box 1). The current data should also be amended, for example, with a dissection of temporal and seasonal variation at global scale, which could be achieved by simultaneously and repeatedly collecting samples of the global ocean. To this end, initiatives of crowd-sourced research are already on the horizon (www.oceansamplingday.org). The increasing quantity, quality and resolution of such data will make it possible to address global-scale phenomena, and by integrating molecular data, to test constraints on biodiversity, dispersal and evolution at various spatial and temporal scales, for example. We anticipate that with advances in ‘omics’ technologies, deciphering the features that are consistent within and across Earth's ecosystems as well as the mechanisms that drive them over seasonal and evolutionary time scales has now become a little more science than just fiction.

**Box 1 *Tara* Oceans: from parts lists towards an understanding of ecosystems**

*Tara* Oceans released a massive amount of primary and derived data along with the publication of their initial results. For example, a data volume of ca. 13 terabytes has already been archived at the EBI (PRJEB402); however, many data types can still not be easily compared as methodological details and context differ.

Due to differing biological features in the different organism classes and due to funding constraints, different methods were applied to capture biodiversity. For example, metagenomics could not be afforded for eukaryotes, since only a very small fraction of the large genomes are protein-coding. Also, because of missing methodological standards, direct comparison of these data is challenging. For example, due to difficulties in delineating species based on molecular data alone, the term operational taxonomic unit (OTU) is commonly used to define a taxonomic group based on sequence similarity of select taxonomic marker genes. However, the 18S and 16S rRNA genes are used for eukaryotes and prokaryotes, respectively, which differ in diversification rates and operational taxonomic definitions. Moreover, as viruses lack any universal genes that could be used for consistent taxonomic classification, long contiguous sequences of assembled viral genomes were used as an alternative approach to quantify viral populations. On the other hand, for studying genetic diversity, similar gene definitions were used for metagenomically characterized prokaryotic genes and metatranscriptomically derived eukaryotic genes. However, sequencing depths, sample numbers, gene lengths, genome sizes and many other parameters are different and need normalization, before sensible comparisons can be made (Table1). Thus, despite a 1,000-fold increase of data over earlier ocean surveys (Rusch *et al*, 2007), the established *Tara* Oceans' resources are only the tip of an iceberg when attempting to collect planetary biodiversity. While representing a promising start to collect the molecular and taxonomic parts lists of the contemporary ocean, *Tara* Oceans has a lot of work ahead to connect these into species interactions and their functional meaning in the context of the environment.
